# First person – Linda Greening

**DOI:** 10.1242/bio.060061

**Published:** 2023-07-20

## Abstract

First Person is a series of interviews with the first authors of a selection of papers published in Biology Open, helping researchers promote themselves alongside their papers. Linda Greening is first author on ‘ Towards an objective measurement of sleep quality in non-human animals: using the horse as a model species for the creation of sleep quality indices’, published in BiO. Linda is a senior lecturer at Hartpury University, currently studying equine sleep behavior specifically, and researching and lecturing more broadly in the field of equine behaviour and welfare.


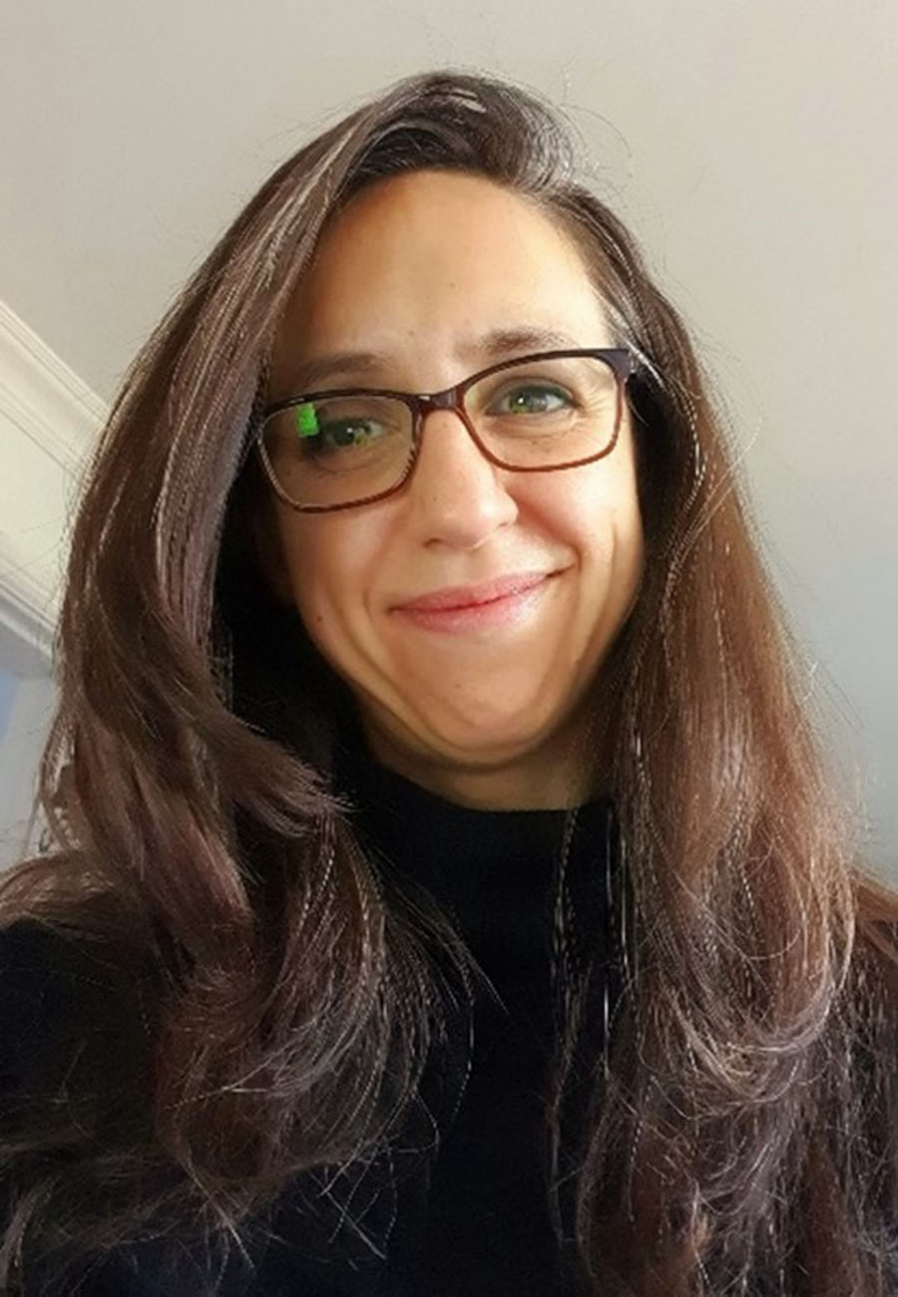


**Linda Greening**

**Describe your scientific journey and your current research focus**

I began my career as a junior lecturer in 2006 and conducted a number of studies in collaboration with students, which we took to conferences as posters or presentations. Early on, these studies were largely focused around equine learning and stereotypic/abnormal repetitive behaviours but in 2011, I supervised a project looking at horse sleep and my interest in the topic grew and continued from there until where I am now. In a small, specialist higher-education institution, resources are tight, and I spent time increasing my knowledge and understanding of equine sleep until I was able to build a strong justification to request funds to invest in equipment for use in teaching and research. The projects conducted as a result of this investment provided provisional data that served as a good platform from which to promote opportunities for collaborations and build bids for external funding.

**Who or what inspired you to become a scientist?**

I wanted to be a vet. I didn't quite make the grades but loved science and wanted to study it at university. I chose an applied biology degree (equine science) to do so. The lack of evidence-informed practice in the equine industry is surprising and I found a number of opportunities to generate new knowledge through science to help the industry progress.

**How would you explain the main finding of your paper?**

Sleep quality tends to be self-reported in human sleep research, which is not possible for non-human animal species. We used the number of awakenings within sleep sequences to devise a way to quantify sleep quality and applied this to an existing data set from which we had previously reported sleep quantity data. Results indicated how sleep quality is different from sleep quantity, but more research is required to understand the outcomes of poor-quality sleep as determined by this metric.

**What are the potential implications of this finding for your field of research?**

The field of equine sleep research is hampered by lack of funding and lack of access to gold-standard technology such as electroencephalography (EEG). Thus, deriving valid and reliable behavioural metrics enable exploration of equine sleep. We believe the indices we developed could be applied to other mammalian species too.

… deriving valid and reliable behavioural metrics enable exploration of equine sleep … the indices we developed could be applied to other mammalian species too.

**Figure:**
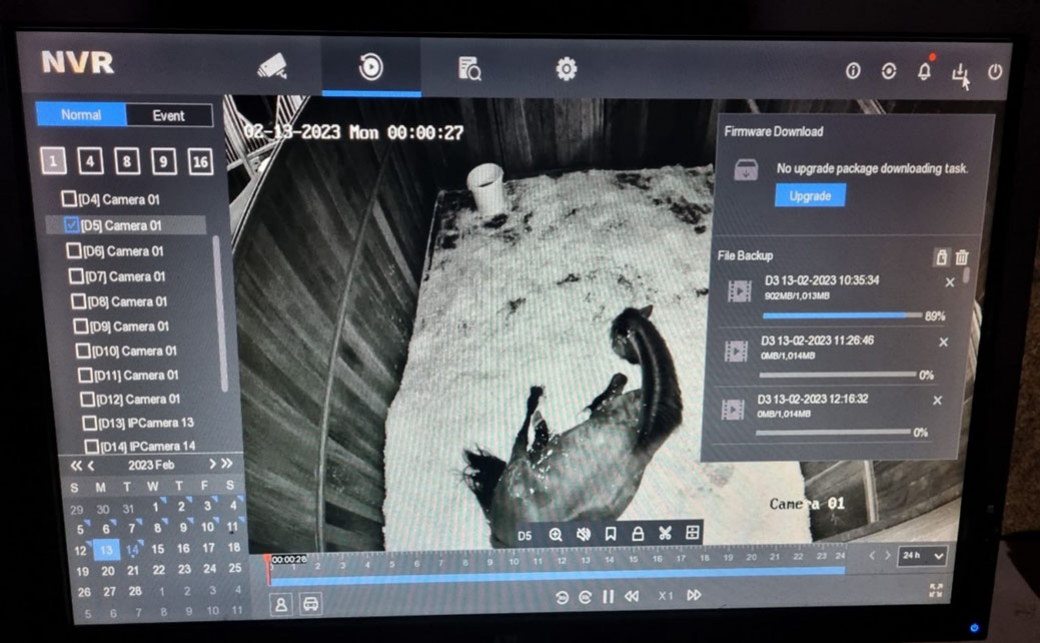
So many hours of data!

**Which part of this research project was the most rewarding?**

The sleep-quality metric was born from interrogation of existing equine EEG data, which visualized just how fragmented equine sleep is. We had been using a blunt tool of cumulative duration of sleep state but in fact, looking at the number of awakenings, duration data may not be entirely accurate. Being able to apply the indices to data and realise they were functional was brilliant and I hope they prove useful for future researchers to consider sleep quality too.

The sleep-quality metric was born from interrogation of existing equine EEG data, which visualized just how fragmented equine sleep is.

**What do you enjoy most about being an early-career researcher?**

There is no fear of getting it wrong!

**What piece of advice would you give to the next generation of researchers?**

Listen carefully and reflect on your conversations with established researchers, think about how you can adapt practice and process to suit your needs and skills. Look for opportunities to up-skill at every juncture.

**What's next for you?**

I am part of a bid writing team looking at validating behavioural work with EEG data and then training AI to interpret data – it is exciting, and will increase efficiency of research in this field to encourage others to engage with it, so fingers crossed we are successful!
